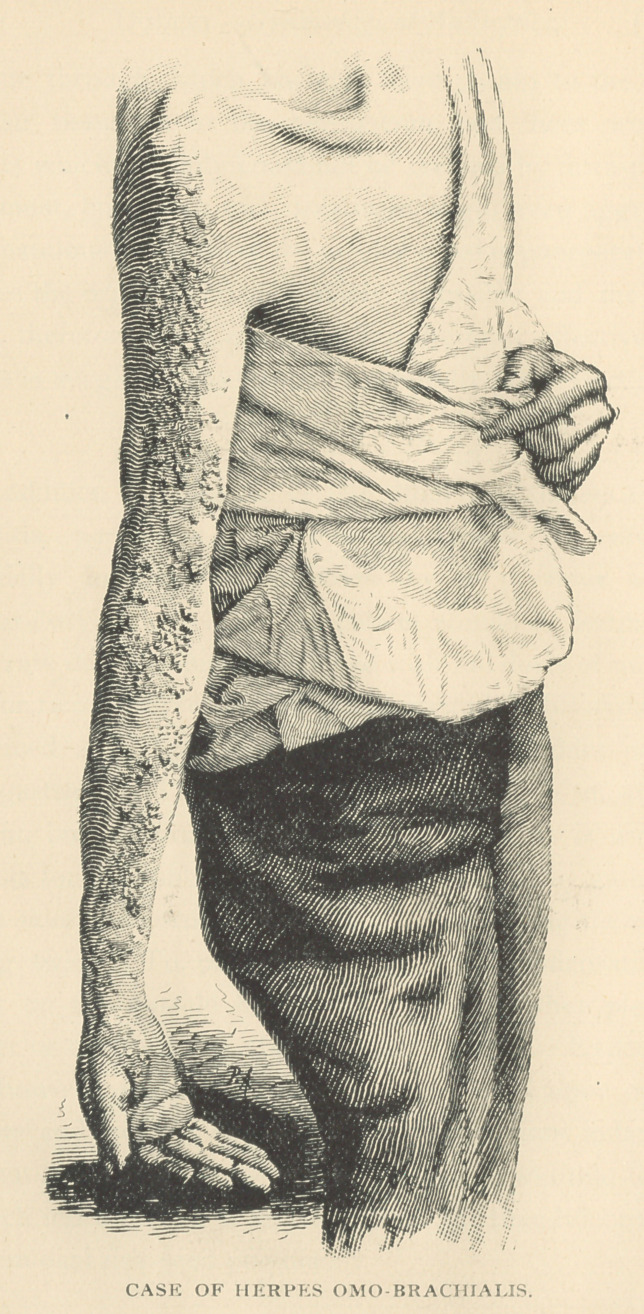# Case of Herpes Omo-Brachialis

**Published:** 1887-03

**Authors:** James I. Tucker

**Affiliations:** 52 Douglas avenue; Chicago


CASE OF HERPES OMO-BRACHIALIS.

### CASE OF HERPES OMO-BRACHIALIS. *By* JAMES I. TUCKER, A.M., M.D., *Chicago.*

[Read before the Chicago Medical Society, March 21st.]

Herpes is one of that class of skin diseases which are especially interesting because, though characterized by a distinctly defined cutaneous eruption, they are not, properly speaking, diseases of the skin. I believe that it is universally conceded by physicians that they belong to the neurological group, and that the peripheral phenomena which they exhibit are traceable to altered trophic centres residing in the ganglion or to disturbed trophic innervation. This fact has been repeatedly observed in frontal zoster, and is doubtless equally true of zoster appearing in other regions besides those presided over by the ganglion of Gasser and branches of the fifth pair.

The case to which I wish to call your attention is the following. Mr. J. P., aged 36 years, had for many years been an invalid owing to indiscretions in his youth. He had been very intemperate in the use of spirituous liquors and sexually inconstant; he contracted syphilis and went through all the several stages of this disease, which was aggravated by the injudicious treatment of a border physician. He was one of a family every member of which had been afflicted with some form of nervous disorder, a family so peculiar in this respect that I made the phenomena exhibited by them the theme of a paper which I read before this society a few months ago and which I entitled "Undiagnosable Maladies." He came first under my professional care about five years ago. He was then much emaciated, anæmic, and suffering from a profuse serous diarrhœa and a remarkable effusion of synovial fluid of the knees, the patellæ literally floating in a sea of this fluid. He recovered from this disease and found employment as a proof-reader, and pursued his avocation diligently till about six months ago when he completely broke down and came under my care a second time His habits were now and for a long time had been good in every respect. His principal complaint was a cough—a cough of deep, sepulchral sound, which gave him no rest night or day. I never listened to such a profound and persistent cough before, and it was useless also, for there was no mucus or other foreign matter to be expectorated. Knowing the history of the family as well as I did, and educing no satisfactory evidence of organic disease of lung, heart, liver or kidney, as a temporary makeshift I called it *nervous*. On the 3d of December he came to see me on account of a pain of a rheumatic character in the right shoulder and arm. On December 4th there appeared an eruption consisting of a few vesicles, almost invisible, in clusters on an inflamed base. They crept hour by hour and day by day in quick succession, attended with an almost insufferable sensation of heat, an ignis sacra so intense that according to his description it seemed as if the shoulder and arm were in the state of ebulition and as if, if he had looked at them, each vesicle would have been seen undulating like the rings of a polyp. The vesicular eruption had fully evolved itself by the 9th, and occupied the shoulder and outer aspect of the arm and forearm, crop after crop appearing and following the course of branches of the brachial plexus, notably of the circumflex, the musculo-spiral, the external cutaneous and the median nerves, and terminating in an immense and extremely painful bulla situated upon the ball of the thumb. These vesicles in due course of time passed through the various stages of increase, confluence, maturation, decline and termination. The eruption had ertirely disappeared by December 15th, though the inflamed bases remained distinctly visible. The causalgia ceased, and more remarkable still, the chronic cough disappeared also altogether. The eruption was such an unique example of this rare form of herpes that I took a photograph of it, and have it here to exhibit to you.

To relieve the pain and burning sensation I resorted to hot fomentations and the oleate of cocaine. I found an ointment of the boro-glycerite to be useful. It was of very little use to employ opiates and anodynes, for he seemed to be proof against then. He was very nervous and sleepless, but morphia in large doses had very little effect upon him; and one time, during his former illness, I recollected he took two hundred and forty grains of chloral hydrate (of his own accord) at a single dose, with no effect except to make him a little delirious! In treating him I could not but think that one of the spinal ganglia had been affected by the old syphilitic virus, and accordingly made use of the iodide of potassium.

In this case several problems present themselves for solution:

1. What may have been the relation existing between the cough and the eruption?

2. Why did the cough cease upon the appearance of the the eruption?

3. May the ganglion have been the seat of a tertiary syphilitic alteration and undergone a metastasis from the ganglion to the skin, and culminated in a morbid peripheral trophic and sensitive phenomenon?

52 Douglas avenue.

## Figures and Tables

**Figure f1:**